# The risks for major psychiatric disorders in the siblings of probands with major depressive disorder

**DOI:** 10.1038/s41380-024-02650-1

**Published:** 2024-07-07

**Authors:** Sang Jin Rhee, Linda Abrahamsson, Jan Sundquist, Kristina Sundquist, Kenneth S. Kendler

**Affiliations:** 1 Department of Neuropsychiatry, Seoul National University Hospital, Seoul, South Korea; 2 Center for Primary Health Care Research, Lund University, Malmö, Sweden; 3 Department of Family Medicine and Community Health, Department of Population Health Science and Policy, Icahn School of Medicine at Mount Sinai, New York, NY USA; 4 Virginia Institute for Psychiatric and Behavioral Genetics, Virginia Commonwealth University, Richmond, VA USA; 5 Department of Psychiatry, Virginia Commonwealth University, Richmond, VA USA

**Keywords:** Genetics, Depression

## Abstract

Using a case-controlled study including siblings of major depression (MD) and control probands, born 1970–1990 and followed through 2018, we sought to clarify the degree to which the familial liability to MD is reflected in its clinical features, and the pattern of psychiatric disorders at elevated risk in the siblings of MD probands. The study population included full-siblings of 197,309 MD and matched 197,309 control probands. The proband-sibling tetrachoric correlation of for MD was +0.20. Both linear and quadratic effects of younger AAO and number of episodes significantly increased the risk of MD in siblings. Male sex, anxiety disorder, alcohol use disorder (AUD), inpatient treatment, psychotic symptoms, severity, and antidepressant prescription in MD probands increased the risk of MD in siblings. Cox proportional hazard models (hazard ratios, 95% CI) revealed a significantly increased risk of attention deficit hyperactivity disorder (1.82, 1.76–1.88), generalized anxiety disorder (1.79, 1.74–1.85), bipolar disorder (1.78, 1.70–1.85), MD (1.74, 1.72–1.76), obsessive-compulsive disorder (1.72, 1.65–1.80), phobic anxiety disorder (1.71, 1.65–1.76), and panic disorder (1.68, 1.64–1.72) in MD co-siblings. The HRs for AUD (1.64, 1.60–1.68), post-traumatic stress disorder (1.62, 1.59–1.66) were modestly lower, and the lowest was seen for schizophrenia (1.42, 1.30–1.54). The overall pattern of increased risk of these disorders was similar in reared-apart half-siblings and cousins of MD probands. Our findings suggest that MD is familial, and a range of important clinical factors predict its familial liability. The familial liability to MD, mostly due to genetic factors, is shared with a broad range of psychiatric disorders.

## Introduction

Major depression (MD), a common and often impairing disorder [1], is familial with twin studies showing familial aggregation largely due to genetic factors [2, 3]. Among depressed patients, early age at onset (AAO) [3–9], recurrence [3, 4, 6, 7, 10], and comorbidity with anxiety disorders (AD) or alcohol use disorder (AUD) [4, 11, 12] is associated with higher rates of MD in relatives.

However, the familial co-aggregation of MD with other psychiatric disorders including AD [13–16], obsessive-compulsive disorder (OCD) [17], bipolar disorder (BD) [9, 16, 18], attention deficit hyperactivity disorder (ADHD) [13], post-traumatic stress disorder (PTSD) [19], AUD [20], and schizophrenia (SZ) [21] has been reported. While modest levels of sharing of familial risk is widespread among psychiatric disorders (e.g. [22–24]), the genetic liability to MD may have particularly wide-spread effects [25].

Prior studies of the familial aggregation of MD have largely utilized personal interviews in modest to moderate sized samples [3, 15]. High-quality nationwide registries now permit such investigations with much larger sample size and more precise results. Primary care data, where the majority of MD cases are registered, is particularly important for such investigations [26].

To further understand critical features of the genetic epidemiology of MD, we conducted a study of siblings of MD cases and matched controls in Sweden where primary care data is available. Siblings are common and the only class of first-degree relatives of the same generation, thereby controlling for cohort effects. In this report, we try to address the following questions:

1. What is the degree of aggregation of MD within siblings and how is this influenced by the sex of the proband?
2. How is MD risk in siblings affected by two indices of severity of MD in the proband -- age at onset and levels of recurrence? Can familial liability to MD be reflected in other clinical features?
3. How specific is the familial risk for MD as assessed by levels of co-aggregation of these disorders in siblings of MD probands: generalized anxiety disorder (GAD), panic disorder (PD), phobic anxiety disorder (phobia), OCD, BD, ADHD, PTSD, AUD, and SZ?
4. Through an examination of patterns of disorder co-aggregation in reared-apart half-siblings and cousins, can we clarify the degree to which our full-sibling findings result from genetic versus shared environmental factors?

## Subjects and methods

We collected information on individuals from Swedish population-based registers with national coverage (Supplementary Table 1), linking each person’s unique personal identification number which, to preserve confidentiality, was replaced with a serial number by Statistics Sweden. This research was conducted in accordance with the Declaration of Helsinki. We secured ethical approval for this study from the Regional Ethical Review Board in Lund and no participant consent was required (No. 2008/409 and later amendments).

Psychiatric diagnoses of MD, as well as GAD, PD, Phobia, AD, OCD, BD, ADHD, PTSD, AUD, and SZ, were searched for using the Swedish Hospital Discharge Register, Outpatient Care Register, almost nationwide primary care data, the Swedish Prescribed Drug Register, the Swedish Cause of Death Register, the Swedish Criminal Register, and the Swedish Suspicion Register. Usage of antidepressants were searched for in the Swedish Prescribed Drug Register. For details of diagnostic/variable identification, see Supplementary Table 2.

Our source population consisted of all individuals born in Sweden 1970–1990, having Swedish-born parents; *n* = 1,783,302. Immigrants were excluded because information on family members is often lacking, and their access to psychiatric care might vary by disorder for cultural reasons. We also excluded *n* = 22,736 individuals dying before the age of 25 and *n* = 63,962 individuals emigrating before age 25. The age of 25 was used to ensure we had follow-up time to enable retrieval of psychiatric diagnoses from the registries.

The 1,696,604 individuals left were grouped into 1,015,248 unique parental pairs. Out of these, we were interested in the 522,202 unique parental pairs (made up by a total of *n* = 1,037,005 parents) where we could find a sibship size of at least two full-siblings. The sibships were made up of a total of 1,203,558 individuals. Case probands were defined as any of these individuals having MD, but not BD or SZ, rendering *n* = 197,309. Each case proband was matched with one control proband, which was drawn from the group without any diagnosis of BD or SZ, *n* = 1,182,956. Control probands were matched by sex, and age by applying incidence density sampling, i.e. we ensured that the matched control proband had follow-up time and no MD diagnosis at least until the same age as the MD diagnosis of the case proband. The siblings of the probands were not screened for BD and SZ. Apart from the type of matching used throughout this study, five other types were performed to determine their influence on the results (Supplementary Table 3).

Amongst the previously described group of *n* = 1,696,604 individuals, we also searched for reared-apart half-siblings and first cousins. In this group, 266,559 had at least one half-sibling (158,399 had at least one maternal half-sibling and 161,654 had at least one paternal half-sibling) and 1,398,416 had at least one cousin. In total, we detected 227,688 pairs of half-siblings and 3,357,273 pairs of cousins. Amongst the half-sibling pairs 102,668 were reared-apart (not living together during ages 0–15). The information on rearing were taken from household identification number (Population and Housing Census; years 1970–1985) and from family identification (Total Population Register; year 1986 and onwards). In the reared-apart half-siblings group, and in the cousin group, we detected 30,117 and 235,361 case probands, respectively, diagnosed with MD but not BD or SZ. Matching of control probands was made in the same manner as for full-siblings, within the *n* = 102,668 half-sib and *n* = 1,130,355 cousin pairs.

In full-siblings to case probands and control probands, we examined the impact of lifetime proband diagnoses of MD (i.e. case/control status of proband) on time to first diagnosis of MD, GAD, PD, Phobia, OCD, BD, ADHD, PTSD, AUD and SZ in siblings, from the age of ten, using a multivariable Cox proportional hazards model with age of sibling as time scale, stratified on the matched pairs of probands. The sibling was followed until time point of diagnosis, study end of 2018-12-31, death or emigration, whichever occurred first. To take into account dependency within families, standard errors were obtained from robust variance estimation. We adjusted for sex and birth year of the siblings in the models. To study the effect of having an MD-affected sibling, we adapt to the Cox model setting, a proband correction proposed by Weinberg [27]. In the procedure, we correct for the fact that we only have information on sibships whom at least one MD-affected sibling. When applying the correction, we allow an individual affected with MD, to be counted as proband, once for each co-sibling, and to be counted as co-sibling, once for each sibling affected with MD. Each unique proband-sibling pair is one observation in data. The same type of modeling was applied to reared-apart half-siblings and cousins. Apart from studying the HR of proband MD on the 10 diagnoses mentioned above, we also studied, in full-siblings, the effect of proband MD AAO, measured by age at first MD registration, on the 10 diagnoses. We did this by including an interaction term in our main model between case proband and proband younger AAO, the latter as a continuous variable in units of 5 years. We included AAO as a linear term, however when specifically studying MD as outcome, we also included AAO as both a linear and a quadratic term. The same type of modeling was also applied for studying the effect of number of proband MD episodes (a discrete numeric variable) in the registries. An episode was defined as a registration of MD in any of the medical registries, without any previous registration within the last 90 days. Specifically for MD in sibling as outcome, we also performed several further analyses: Using the main Cox model, we included a multiplicative interaction term between case proband and variables of proband sex, AD in proband, AUD in proband, MD in proband diagnosed at least once in inpatient care, psychotic MD in proband, antidepressants prescribed to proband between July 2005 and 2018, and MD severity in proband, one variable at a time. For the MD severity in proband, we included two different interaction terms in the model: case proband with moderate MD in proband and severe MD in proband (using mild MD as the reference). Severity of MD was defined as the highest degree of MD severity ever found in the medical registries. For the MD diagnosis in co-sibling as outcome, we also calculated tetrachoric correlations between actual case/control status of proband with co-sibling outcome, as well as ever having MD in proband with co-sibling outcome. Tetrachoric correlations were calculated as this measure of association as it is easy to interpret in a genetic-epidemiologic context and is relatively insensitive to changes in base rates. In the main Cox model for full-siblings, sensitivity analyses were performed to account for proband comorbidities (Supplementary Table 7).

Levels of significance of 0.05, 0.01, 0.001 and 0.0001 were marked in tables. Bonferroni correction procedures for multiple testing, using a significance level of 0.05, were applied to tables including within-outcome comparisons of the proband MD effect. Data analysis was conducted from June 1, 2023, to September 21, 2023. Statistical analyses were performed using R, version 4.2.1 [28] (Supplementary Table 4) and SAS, version 9.4 [29].

## Results

### Descriptive statistics

Table 1 provides the descriptive results of probands and their full-siblings. Of 197,309 MD probands, 63.2% were female, and the mean (SD) AAO of MD was 30.2 (6.8). Eventually, 10.4% of 197,309 matched control probands were diagnosed with MD between ascertainment and the end of follow-up. There was a median (IQR) of one (1–2) sibling for both MD and control probands. Prevalence rates of MD in siblings of MD and control probands were 25.7 and 16.4%, respectively. The prevalence rates of other psychiatric disorders ranged from 0.4% for SZ to 8.8% for PTSD in siblings of MD probands, which were all higher than in siblings of control probands.

**Table 1:** Demographics of the total study population.

	MD Proband	Control Proband
Proband		
Sample size, No.	197,309	197,309
Female, %	63.2	63.2
Birth year, mean (sd)	1980.5 (5.7)	1978.0 (5.4)
MD prevalence, %	100	10.4
Age at onset of MD, mean (sd)	30.2 (6.8)	35.0 (5.7)
No. of siblings, median (IQR)	1 (1–2)	1 (1–2)
Sibling		
Sample size, No.	289,357	284,408
Female, %	48.7	48.3
Birth year, mean (sd)	1980.4 (5.7)	1979.0 (5.6)
MD prevalence, %	25.7	16.4
Age at onset of MD, mean (sd)	29.4 (6.8)	31.1 (6.8)
GAD prevalence, %	4.4	2.6
PD prevalence, %	6.9	4.2
Phobia prevalence, %	3.7	2.1
OCD prevalence, %	2.0	1.2
BD prevalence, %	2.2	1.3
ADHD prevalence, %	4.7	2.5
PTSD prevalence, %	8.8	5.8
AUD prevalence, %	5.2	3.4
SZ prevalence, %	0.4	0.3

*MD* major depression, *sd* standard deviation, *IQR* interquartile range, *GAD* generalized anxiety disorder, *PD* panic disorder, *Phobia* phobic anxiety disorder, *OCD* obsessive-compulsive disorder, *BD* bipolar disorder, *ADHD* attention deficit hyperactivity disorder, *PTSD* post-traumatic stress disorder, *AUD* alcohol use disorder, *SZ* schizophrenia.

### Co-aggregation of MD in siblings

The risk for MD of siblings was significantly higher in MD than control probands (HR = 1.74, 95% CI = 1.72–1.76). The tetrachoric correlation of case/control-status in probands vs MD diagnosis in siblings was +0.20 (95% CI = 0.20–0.21). The HRs and tetrachoric correlations were similar despite varying sampling methods of control probands (Supplementary Table 3).

**Fig. 1:**
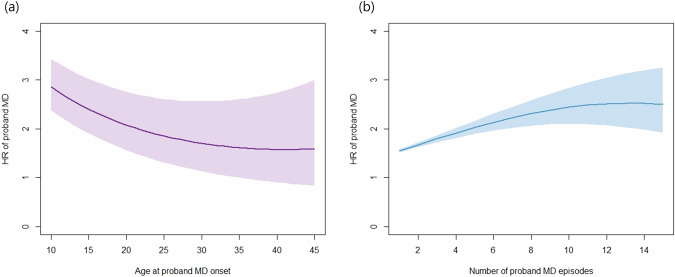
Hazard Ratio for MD in Siblings. The hazard ratio with standard errors for MD in sibling as a function of the **a** age at onset, and **b** number of episodes of MD proband. Age at onset measured on a time scale of 5 years. Both linear and quadratic effects are reflected. HR hazard ratio, MD major depression.

The risk of MD in siblings as a function of the presence or absence of the clinical features in MD probands is summarized in Table 2. For younger AAO and greater number of episodes, linear and quadratic effects were both significant. As depicted in Fig. 1a, earlier AAO predicts a higher HR, and as AAO increases the HR declines and flattens after age 35. Considering recurrence (Fig. 1b), the HR increases as the number of episodes increase, but gradually flattens and reaches a plateau after 10–12 episodes. The risk of MD in siblings was significantly higher when the proband was male. Finally, AD and AUD comorbidity, inpatient treatment, psychotic symptoms, severity, and antidepressants prescription in the MD proband all significantly increased risk for MD in their siblings.

**Table 2:** Individual clinical features of MD proband, and their association with risk of MD in siblings.

	HR	95% CI	*P*-value^a^
Younger age at onset^b^			
Linear effect only	1.07	(1.06–1.08)	<0.0001*
Linear and quadratic effect	Linear: 1.29 Quadratic: 1.02	(1.20–1.37) (1.01–1.02)	<0.0001* <0.0001*
Number of episodes			
Linear effect only	1.05	(1.05–1.06)	<0.0001*
Linear and quadratic effect	Linear: 1.09 Quadratic: 0.997	(1.08–1.10) (0.996–0.998)	<0.0001* <0.0001*
Sex			
Proband male	1.08	(1.05–1.11)	<0.0001*
Comorbidity			
AD	1.21	(1.19–1.24)	<0.0001*
AUD	1.26	(1.21–1.32)	<0.0001*
Inpatient	1.16	(1.12–1.21)	<0.0001*
Psychotic depression	1.20	(1.07–1.35)	0.002*
Severity			
Moderate (Ref: Mild)	1.18	(1.13–1.23)	<0.0001*
Severe (Ref: Mild)	1.23	(1.17–1.31)	<0.0001*
Antidepressants	1.27	(1.23–1.31)	<0.0001*

^a^Based on the interaction between clinical features and MD in sibling, controlled for sex and birth year of the siblings. *Significant at Bonferroni corrected levels of *P* < 0.05/17 = 0.0029.

^b^Younger age at onset is measured as a continuous variable, in five-year units.

*MD* major depression, *AD* anxiety disorder, *AUD* alcohol use disorder, *HR* hazard ratio, *CO* confidence interval.

### Co-aggregation of major psychiatric disorders in siblings

**Fig. 2:**
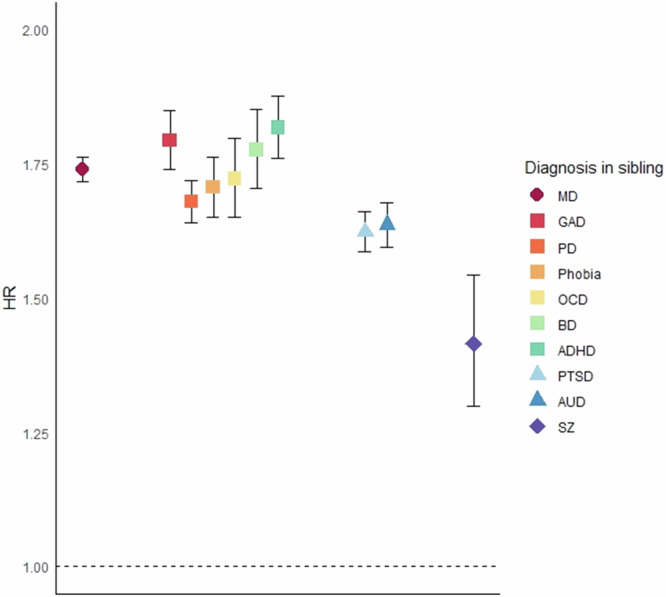
The impact of major depression (MD) proband status on the hazard ratio in siblings for MD, anxiety disorders, obsessive-compulsive disorder, bipolar disorder, attention deficit hyperactivity disorder, post-traumatic stress disorder, alcohol use disorder, and schizophrenia. Analyses was controlled for sex and birth year of the siblings. Diagnosis in siblings were all measured from age 10 and onwards for reasons of stability. HR hazard ratio, MD major depression, GAD generalized anxiety disorder, PD panic disorder, Phobia phobic anxiety disorder, OCD obsessive- compulsive disorder, BD bipolar disorder, ADHD attention deficit hyperactivity disorder, PTSD post-traumatic stress disorder, AUD alcohol use disorder, SZ schizophrenia.

Figure 2 and Supplementary Table 5 shows the HRs (95% CIs) of 10 major psychiatric disorders in the siblings of MD vs control probands. The HRs were significantly increased for all major psychiatric disorders, the highest was for ADHD (1.82, 1.76–1.88), followed by GAD (1.79, 1.74–1.85), BD (1.78, 1.70–1.85), MD (1.74, 1.72–1.76), OCD (1.72, 1.65–1.80), Phobia (1.71, 1.65–1.76) and PD (1.68, 1.64–1.72). The HRs for PTSD (1.62, 1.59–1.66) and AUD (1.64, 1.60–1.68) were modestly lower, and the lowest was for SZ (1.42, 1.30–1.54).

**Fig. 3:**
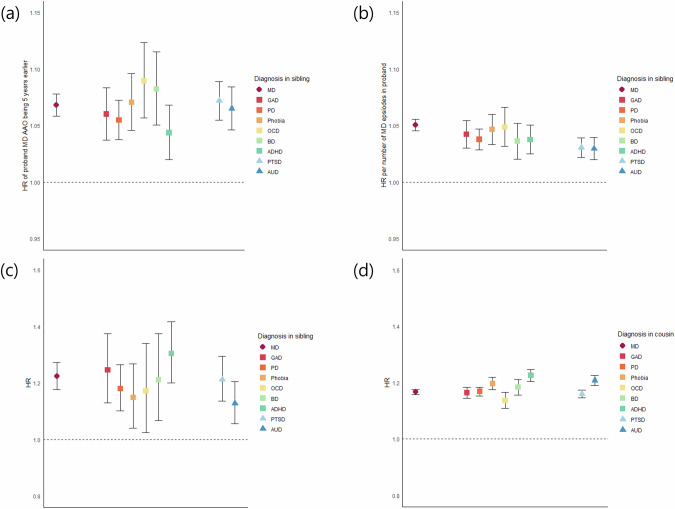
Impact of Clinical Features of MD on risk patterns in siblings and Proband MD on risk patterns in half-siblings and cousins. Linear effects of **a** younger age at onset and **b** number of episodes of MD probands on the risk of major psychiatric disorder in siblings. The impact of MD on the risk of psychiatric disorders in **c** reared-apart half-siblings and in **d** cousins. The psychiatric disorders that were analyzed were MD, anxiety disorders, obsessive-compulsive disorder, bipolar disorder, attention deficit hyperactivity disorder, post-traumatic stress disorder, and alcohol use disorder. The results for schizophrenia were not presented as the sample size was too small for the results to be informative. Analyses was controlled for sex and birth year of the siblings. Only linear effect was analyzed to enable comparison between psychiatric disorders. Younger age at onset was measured as a continuous variable, in five-year units. Diagnosis in siblings and cousins were all measured from age 10 and onwards for reasons of stability. HR hazard ratio, AAO age at onset, MD major depression, GAD generalized anxiety disorder, PD panic disorder, Phobia phobic anxiety disorder, OCD obsessive- compulsive disorder, BD bipolar disorder, ADHD attention deficit hyperactivity disorder, PTSD post-traumatic stress disorder, AUD alcohol use disorder.

Figure 3a, b and Supplementary Table 6 shows the linear effects of AAO and number of episodes in MD cases on the HRs of our 10 disorders. The effect of younger AAO increasing HRs were similar and was significant for all 10 disorders except for SZ. The number of episodes nearly equally predicted a higher risk in all of these disorders. Sensitivity analyses revealed that the probands’ psychiatric comorbidity was not a major factor in increasing the risk of other psychiatric disorders in siblings of MD probands, as the HR dropped only modestly when controlling for comorbidity using two different methods (Supplementary Table 7).

### Analysis of reared-apart half-siblings and cousins

Finally, the impact of MD on the risks of major psychiatric disorders in reared-apart half-siblings and cousins were analyzed and depicted in Fig. 3c, d. When compared with full-siblings, the overall patterns were similar. The differences were that the HRs were lower in reared-apart half-siblings and cousins, and the risk for SZ was no longer significant in reared-apart half siblings.

## Discussion

In our study, we sought to address four major questions in MD cases through treatment facilities and control probands, which we review in turn.

First, we found sibling resemblance for MD with a significant HR of 1.7. Although this is lower than previous studies reporting a 2 to 3 times higher risk in siblings [9, 30], the tetrachoric correlation (+0.20) was similar to that found in dizygotic twins in previous twin studies [2, 31, 32] and what would be expected given a heritability of MD of ~0.40 [3]. The lower HR likely results from the relatively high rates of MD in the Swedish population including our primary care data. Interestingly, our study showed a modest excess risk for MD in siblings of male versus female MD probands. This is consistent with predictions from a multiple threshold model given the lower prevalence of MD in men versus women [33] but has been rarely reported elsewhere. A recent finding from the Swedish registry also showed that affected males had higher family genetic risk scores than affected females [34].

Second, sibling risk for MD was, consistent with most [4–9] but not all [10, 12, 30] prior familial studies, associated with both AAO and high recurrence rates. The size of our sample permitted us to show significant quadratic effects for AAO and, for the first time to our knowledge, recurrence, with the effects largely disappearing at AAO over 35 and over 10–12 recurrences. Previous studies have suggested that the etiology of childhood-onset depression differs and are more strongly genetically influenced than adult-onset depression [35]. It is also known that early-onset MD is associated with a higher familial risk of MD, while late-onset MD is associated with an increased familial risk of vascular disease [36]. The effect of other environmental factors not shared by siblings, including chronic diseases and lifestyle risk factors, probably are more prominent in late-onset MD [8]. Furthermore, in accordance with previous literature [3, 4, 6], severity indices increased the risk in siblings for MD probands, in which our study included inpatient treatment, symptoms of psychosis, clinical severity as indexed by ICD criteria, and treatment with antidepressants. Finally, also in line with previous reports [4, 12], we found risk for MD in siblings was increased by proband comorbidity with AD and AUD.

Third, the pattern of risks for a range of other disorders in the siblings of MD probands suggested that the familial liability to MD was relatively nonspecific. The risks for the disorders examined could be usefully divided into three major groups: The first group was made up of internalizing disorders (GAD, PD, Phobia, and OCD), other mood disorders (BD), and ADHD. For these disorders, the HR in siblings of MD probands was statistically indistinguishable from that found for MD itself. The second group was PTSD and AUD where the HRs in siblings were modestly lower than that seen for MD. The third group, consisting only of SZ, had HRs in siblings appreciably lower than that seen for MD.

The nonspecific risk of MD has been suggested in a meta-analysis of offspring [13], and familial genetic risk scores [25]. However, there are opposite results, including a study from Germany where offspring of MD cases showed specific vulnerability to MD compared to AD [15]. MD has been historically connected with BD in Kraepelin’s category of manic-depressive illness [37], and is genetically close to internalizing disorders like AD [14, 24]. Our study expands previous results in the genetic epidemiology of MD, that MD reflects a broad vulnerability to many common forms of psychopathology. These nonspecific risks are supported by molecular studies showing genetic dispositions to MD and other psychiatric disorders substantially overlap [22, 23]. Importantly, we were able to replicate the broad nature of the familial liability to MD by an independent within MD-sibship design, showing that earlier AAO and higher levels of recurrence predicted a higher risk for various psychiatric disorders at a level similar to that seen for the risk of MD itself.

Fourth, as siblings share both genes and environments, we cannot, through the study of siblings alone, discriminate the degree to which it is due to genetic factors. We therefore repeated our analyses in reared-apart half-siblings and cousins whose resemblance can, with some confidence, be attributed largely or entirely to genetic effects. The general patterns were similar with those in full-siblings although HRs are all substantially lower in half-siblings and cousins, given the reduction in genetic relatedness, and the confidence intervals are larger in half-siblings and smaller in cousins, given the differences in sample sizes compared to full siblings. From these findings, we can conclude that the pattern of risks for major psychiatric disorders in the siblings of our MD probands was predominantly due to genetic effects.

## Limitations

Our results should be interpreted in the context of three important methodological concerns. First, the validity of our study is dependent on the quality of diagnoses in the Swedish national registries, which have been well supported for diagnoses for BD, SZ, and OCD [38–41]. The validity of MD and AD diagnoses is supported by their prevalence, sex ratio, sibling and twin correlations, and associations with known psychosocial factors [4, 26]. The validity of ADHD diagnosis has been supported by previous studies including a close association with stimulant prescriptions [42]. The validity of AUD is supported by high rates of concordance observed across ascertainment methods [43, 44] and patterns of resemblance in relatives [45]. Second, the absence of shared environment effects does not mean that the influences of siblings are irrelevant. We cannot rule out that clinicians knew the family history of their siblings, and that their knowledge influenced their diagnoses. However similar nonspecific patterns seen in reared-apart half siblings and cousins shows that these effects are likely to be minimal. Third, we used high-quality registers, including primary care, but could not ascertain cases in individuals who never presented for treatment. Therefore, extrapolation to populations who do not seek treatment is limited. Fourth, given that this study was conducted in the Swedish population, generalization to other ethnicities may be limited.

## Conclusions

The results from our large registry-based sample of Swedish siblings support that MD is familial, and that certain clinical features including AAO, recurrence, clinical severity, and presence of psychosis, can meaningfully predict the risk of illness in siblings. Importantly, we show that the increasing risk of illness in relatives of MD probands asymptotes out at older AAO and high levels of recurrence. Familial liability to MD is not highly specific but rather is shared with a broad range of mood and anxiety disorders as well as OCD and PTSD, and, to a more modest degree, ADHD, AUD, and SZ. Results in our analysis of reared-apart half-siblings and cousins suggest that most of the aggregation of MD and other major psychiatric disorders we observed in siblings results from genetic factors. These results have implications for our understanding of the nature of familial/genetic liability to MD, how it is reflected in the clinical presentation of depressive illness and its impact on risk for a wide range of other common psychiatric disorders.

## Supplementary information

Supplemental Material

## Data Availability

The nationwide register data used in this paper cannot be shared openly by the researchers due to legal reasons related to people’s integrity. They could, however, be obtained from the Swedish authorities that are referred to in the methods section in the manuscript. Kristina Sundquist MD PhD had full access to all the data in the study and takes responsibility for the integrity of the data and the accuracy of the data analysis.

## References

[1] GBD 2019 Mental Disorders Collaborators. Global, regional, and national burden of 12 mental disorders in 204 countries and territories, 1990-2019: a systematic analysis for the Global Burden of Disease Study 2019. Lancet Psychiatry. 2022;9:137–50. 10.1016/s2215-0366(21)00395-3. 35026139 10.1016/S2215-0366(21)00395-3 PMC8776563

[2] Kendler KS, Gatz M, Gardner CO, Pedersen NL. A Swedish national twin study of lifetime major depression. Am J Psychiatry. 2006;163:109–14. 10.1176/appi.ajp.163.1.109. 16390897 10.1176/appi.ajp.163.1.109

[3] Sullivan PF, Neale MC, Kendler KS. Genetic epidemiology of major depression: review and meta-analysis. Am J Psychiatry. 2000;157:1552–62. 10.1176/appi.ajp.157.10.1552. 11007705 10.1176/appi.ajp.157.10.1552

[4] Kendler KS, Ohlsson H, Lichtenstein P, Sundquist J, Sundquist K. The genetic epidemiology of treated major depression in Sweden. Am J Psychiatry. 2018;175:1137–44. 10.1176/appi.ajp.2018.17111251. 30021458 10.1176/appi.ajp.2018.17111251

[5] Kendler KS, Gatz M, Gardner CO, Pedersen NL. Age at onset and familial risk for major depression in a Swedish national twin sample. Psychol Med. 2005;35:1573–9. 10.1017/s0033291705005714. 16219115 10.1017/S0033291705005714

[6] Kendler KS, Gatz M, Gardner CO, Pedersen NL. Clinical indices of familial depression in the Swedish Twin Registry. Acta Psychiatr Scand. 2007;115:214–20. 10.1111/j.1600-0447.2006.00863.x. 17302621 10.1111/j.1600-0447.2006.00863.x

[7] Kendler KS, Ohlsson H, Bacanu S, Sundquist J, Sundquist K. Differences in genetic risk score profiles for drug use disorder, major depression, and ADHD as a function of sex, age at onset, recurrence, mode of ascertainment, and treatment. Psychol Med. 2023;53:3448–60. 10.1017/s0033291721005535. 35098912 10.1017/S0033291721005535 PMC10863503

[8] Wium-Andersen MK, Dalgaard Villumsen M, Wium-Andersen IK, Jørgensen MB, Hjelmborg JB, Christensen K, et al. Familial risk and heritability of depression by age at first diagnosis in Danish twins. Acta Psychiatr Scand. 2020;142:446–55. 10.1111/acps.13238. 33010028 10.1111/acps.13238

[9] Li X, Sundquist K, Hemminki K, Sundquist J. Familial risks for depression among siblings based on hospitalizations in Sweden. Psychiatr Genet. 2008;18:80–4. 10.1097/YPG.0b013e3282f08ac9. 18349699 10.1097/YPG.0b013e3282f08ac9

[10] Fernandez-Pujals AM, Adams MJ, Thomson P, McKechanie AG, Blackwood DH, Smith BH, et al. Epidemiology and heritability of major depressive disorder, stratified by age of onset, sex, and illness course in generation Scotland: Scottish family health study (GS:SFHS). PLoS ONE. 2015;10:e0142197. 10.1371/journal.pone.0142197. 26571028 10.1371/journal.pone.0142197 PMC4646689

[11] Kendler KS, Ohlsson H, Sundquist J, Sundquist K. Impact of comorbidity on family genetic risk profiles for psychiatric and substance use disorders: a descriptive analysis. Psychol Med. 2023;53:2389–98. 10.1017/s0033291721004268. 37310304 10.1017/S0033291721004268 PMC10832607

[12] Weissman MM, Merikangas KR, Wickramaratne P, Kidd KK, Prusoff BA, Leckman JF, et al. Understanding the clinical heterogeneity of major depression using family data. Arch Gen Psychiatry. 1986;43:430–4. 10.1001/archpsyc.1986.01800050028003. 3964021 10.1001/archpsyc.1986.01800050028003

[13] Rasic D, Hajek T, Alda M, Uher R. Risk of mental illness in offspring of parents with schizophrenia, bipolar disorder, and major depressive disorder: a meta-analysis of family high-risk studies. Schizophr Bull. 2014;40:28–38. 10.1093/schbul/sbt114. 23960245 10.1093/schbul/sbt114 PMC3885302

[14] Kendler KS, Abrahamsson L, Ohlsson H, Sundquist J, Sundquist K. An extended Swedish adoption study of anxiety disorder and its cross-generational familial relationship with major depression. Am J Psychiatry. 2022;179:640–9. 10.1176/appi.ajp.21111110. 36048482 10.1176/appi.ajp.21111110

[15] Lieb R, Isensee B, Höfler M, Pfister H, Wittchen HU. Parental major depression and the risk of depression and other mental disorders in offspring: a prospective-longitudinal community study. Arch Gen Psychiatry. 2002;59:365–74. 10.1001/archpsyc.59.4.365. 11926937 10.1001/archpsyc.59.4.365

[16] van Dijk MT, Murphy E, Posner JE, Talati A, Weissman MM. Association of multigenerational family history of depression with lifetime depressive and other psychiatric disorders in children: results from the Adolescent Brain Cognitive Development (ABCD) Study. JAMA Psychiatry. 2021;78:778–87. 10.1001/jamapsychiatry.2021.0350. 33881474 10.1001/jamapsychiatry.2021.0350 PMC8060885

[17] Nestadt G, Samuels J, Riddle MA, Liang KY, Bienvenu OJ, Hoehn-Saric R, et al. The relationship between obsessive-compulsive disorder and anxiety and affective disorders: results from the Johns Hopkins OCD family study. Psychol Med. 2001;31:481–7. 10.1017/s0033291701003579. 11305856 10.1017/s0033291701003579

[18] Tsuang MT, Winokur G, Crowe RR. Morbidity risks of schizophrenia and affective disorders among first degree relatives of patients with schizophrenia, mania, depression and surgical conditions. Br J Psychiatry. 1980;137:497–504. 10.1192/bjp.137.6.497. 7214104 10.1192/bjp.137.6.497

[19] Sartor CE, Grant JD, Lynskey MT, McCutcheon VV, Waldron M, Statham DJ, et al. Common heritable contributions to low-risk trauma, high-risk trauma, posttraumatic stress disorder, and major depression. Arch Gen Psychiatry. 2012;69:293–9. 10.1001/archgenpsychiatry.2011.1385. 22393221 10.1001/archgenpsychiatry.2011.1385 PMC3594801

[20] Kendler KS, Lönn SL, Sundquist J, Sundquist K. The actions and interactions of family genetic risk scores for alcohol use disorder and major depression on the risk for these two disorders. Am J Med Genet B Neuropsychiatr Genet. 2022;189:128–38. 10.1002/ajmg.b.32909. 35779072 10.1002/ajmg.b.32909 PMC10016432

[21] Cheng CM, Chang WH, Chen MH, Tsai CF, Su TP, Li CT, et al. Co-aggregation of major psychiatric disorders in individuals with first-degree relatives with schizophrenia: a nationwide population-based study. Mol Psychiatry. 2018;23:1756–63. 10.1038/mp.2017.217. 29112198 10.1038/mp.2017.217

[22] Cross-Disorder Group of the Psychiatric Genomics Consortium. Genomic relationships, novel loci, and pleiotropic mechanisms across eight psychiatric disorders. Cell. 2019;179:1469–82.e11. 10.1016/j.cell.2019.11.020. 31835028 10.1016/j.cell.2019.11.020 PMC7077032

[23] Hindley G, Frei O, Shadrin AA, Cheng W, O’Connell KS, Icick R, et al. Charting the landscape of genetic overlap between mental disorders and related traits beyond genetic correlation. Am J Psychiatry. 2022;179:833–43. 10.1176/appi.ajp.21101051. 36069018 10.1176/appi.ajp.21101051 PMC9633354

[24] Kendler KS, Aggen SH, Knudsen GP, Røysamb E, Neale MC, Reichborn-Kjennerud T. The structure of genetic and environmental risk factors for syndromal and subsyndromal common DSM-IV axis I and all axis II disorders. Am J Psychiatry. 2011;168:29–39. 10.1176/appi.ajp.2010.10030340. 20952461 10.1176/appi.ajp.2010.10030340 PMC3126864

[25] Kendler KS, Ohlsson H, Sundquist J, Sundquist K. The patterns of family genetic risk scores for eleven major psychiatric and substance use disorders in a Swedish national sample. Transl Psychiatry. 2021;11:326. 10.1038/s41398-021-01454-z. 34045441 10.1038/s41398-021-01454-z PMC8160183

[26] Sundquist J, Ohlsson H, Sundquist K, Kendler KS. Common adult psychiatric disorders in Swedish primary care where most mental health patients are treated. BMC Psychiatry. 2017;17:235. 10.1186/s12888-017-1381-4. 28666429 10.1186/s12888-017-1381-4 PMC5493066

[27] Stark A, Seneta E. Wilhelm Weinberg’s early contribution to segregation analysis. Genetics. 2013;195:1–6. 10.1534/genetics.113.152975. 24018765 10.1534/genetics.113.152975 PMC3761293

[28] R: A language and environment for statistical computing (Verstion 4.2.1) [computer program]. Vienna, Austria: R Foundation for Statistical Computing; 2022.

[29] SAS Institute I. SAS/STAT® Online Documentation, Version 9.4. Cary, N.C.: SAS Institute, Inc. In:2012.

[30] Gronemann FH, Jacobsen RK, Wium-Andersen MK, Jørgensen MB, osler m, jørgensen tsh. association of familial aggregation of major depression with risk of major depression. JAMA Psychiatry. 2023;80:350–9. 10.1001/jamapsychiatry.2022.4965. 36753297 10.1001/jamapsychiatry.2022.4965 PMC9909579

[31] Kendler KS, Neale MC, Kessler RC, Heath AC, Eaves LJ. A population-based twin study of major depression in women. The impact of varying definitions of illness. Arch Gen Psychiatry. 1992;49:257–66. 10.1001/archpsyc.1992.01820040009001. 1558459 10.1001/archpsyc.1992.01820040009001

[32] Lyons MJ, Eisen SA, Goldberg J, True W, Lin N, Meyer JM, et al. A registry-based twin study of depression in men. Arch Gen Psychiatry. 1998;55:468–72. 10.1001/archpsyc.55.5.468. 9596050 10.1001/archpsyc.55.5.468

[33] Reich T, James JW, Morris CA. The use of multiple thresholds in determining the mode of transmission of semi-continuous traits. Ann Hum Genet. 1972;36:163–84. 10.1111/j.1469-1809.1972.tb00767.x. 4676360 10.1111/j.1469-1809.1972.tb00767.x

[34] Kendler KS, Ohlsson H, Sundquist J, Sundquist K. Selecting cases of major psychiatric and substance use disorders in Swedish national registries on the basis of clinical features to maximize the strength or specificity of the genetic risk. Mol Psychiatry. 2023; 10.1038/s41380-023-02156-2. 10.1038/s41380-023-02156-2 PMC10832579 37414926

[35] Rice F, Harold G, Thapar A. The genetic aetiology of childhood depression: a review. J Child Psychol Psychiatry. 2002;43:65–79. 10.1111/1469-7610.00004. 11848337 10.1111/1469-7610.00004

[36] Kendler KS, Fiske A, Gardner CO, Gatz M. Delineation of two genetic pathways to major depression. Biol Psychiatry. 2009;65:808–11. 10.1016/j.biopsych.2008.11.015. 19103442 10.1016/j.biopsych.2008.11.015 PMC2744314

[37] Kraepelin E. Psychiatrie: ein Lehrbuch für Studierende und Ärzte. J.A. Barth; 1899:2.

[38] Rück C, Larsson KJ, Lind K, Perez-Vigil A, Isomura K, Sariaslan A, et al. Validity and reliability of chronic tic disorder and obsessive-compulsive disorder diagnoses in the Swedish National Patient Register. BMJ Open. 2015;5:e007520. 10.1136/bmjopen-2014-007520. 26100027 10.1136/bmjopen-2014-007520 PMC4480012

[39] Lichtenstein P, Björk C, Hultman CM, Scolnick E, Sklar P, Sullivan PF. Recurrence risks for schizophrenia in a Swedish national cohort. Psychol Med. 2006;36:1417–25. 10.1017/s0033291706008385. 16863597 10.1017/S0033291706008385

[40] Sellgren C, Landén M, Lichtenstein P, Hultman CM, Långström N. Validity of bipolar disorder hospital discharge diagnoses: file review and multiple register linkage in Sweden. Acta Psychiatr Scand. 2011;124:447–53. 10.1111/j.1600-0447.2011.01747.x. 21838734 10.1111/j.1600-0447.2011.01747.x

[41] Ekholm B, Ekholm A, Adolfsson R, Vares M, Osby U, Sedvall GC, et al. Evaluation of diagnostic procedures in Swedish patients with schizophrenia and related psychoses. Nord J Psychiatry. 2005;59:457–64. 10.1080/08039480500360906. 16316898 10.1080/08039480500360906

[42] Giacobini M, Medin E, Ahnemark E, Russo LJ, Carlqvist P. Prevalence, patient characteristics, and pharmacological treatment of children, adolescents, and adults diagnosed With ADHD in Sweden. J Atten Disord. 2018;22:3–13. 10.1177/1087054714554617. 25376193 10.1177/1087054714554617

[43] Kendler KS, Lönn SL, Salvatore J, Sundquist J, Sundquist K. The origin of spousal resemblance for alcohol use disorder. JAMA Psychiatry. 2018;75:280–6. 10.1001/jamapsychiatry.2017.4457. 29417130 10.1001/jamapsychiatry.2017.4457 PMC5885945

[44] Kendler KS, Ji J, Edwards AC, Ohlsson H, Sundquist J, Sundquist K. An extended Swedish national adoption study of alcohol use disorder. JAMA Psychiatry. 2015;72:211–8. 10.1001/jamapsychiatry.2014.2138. 25565339 10.1001/jamapsychiatry.2014.2138 PMC4351126

[45] Prescott CA, Kendler KS. Genetic and environmental contributions to alcohol abuse and dependence in a population-based sample of male twins. Am J Psychiatry. 1999;156:34–40. 10.1176/ajp.156.1.34. 9892295 10.1176/ajp.156.1.34

