# Supplementary material for: The risks for major psychiatric disorders in the siblings of probands with major depressive disorder
Source: Mol Psychiatry. 2024 Jul 7;30(1):69–75. doi: 10.1038/s41380-024-02650-1 (PMC11649559; doi:10.1038/s41380-024-02650-1)
Supplement: Supplementary file 1 — Supplemental Material [file 41380_2024_2650_MOESM1_ESM.docx]

Supplementary – MD sibling study

Table 1 – Description of Registers

*Multi-Generation Register*

The Multi-Generation Register is a register made up of persons who have been registered in Sweden at some time since 1961 and those who were born in 1932 or later. These are called index persons. The register contains connections between index persons and their biological parents. In 2016, about 12 million index persons were included in the register. The Multi-Generation Register is a part of the register system for Total Population Register, where information comes from the National Tax Board. Every year, a new version of the register is created, including new index persons who immigrated or were born during the year. Information from the Multi-Generation Register may be disclosed for research and statistical purposes. For more information, see *Statistics Sweden, Background Facts, Population and Welfare Statistics 2017:2, Multi-generation register 2016. A description of contents and quality*

*National Patient Register*

In the 1960's the National Board of Health and Welfare started to collect information regarding in-patients at public hospitals, the National Patient Register (NPR). Initially it contained information about all patients treated in psychiatric care and approximately 16 percent of patients in somatic care. The register at that time covered six of the 26 county councils in Sweden. In 1984, the Ministry of Health and Welfare together with the Federation of County Councils decided a mandatory participation for all county councils. From 1987, NPR includes all in-patient care in Sweden. Since 2001, the register also covers outpatient doctor visits including day surgery and psychiatric care from both private and public caregivers. For more information, see *https://www.socialstyrelsen.se/en/statistics-and-data/registers/national-patient-register/*

*Primary Care Data*

We also used information from our new Primary Care research dataset including individual-level information on clinical diagnoses from primary health care centers from the following Swedish counties: Blekinge (2009-2018), Dalarna (2005-2018), Gotland (2011-2018), Gävleborg (2010-2018), Halland (2007-2018), Jönköping (2008-2018), Kalmar (2007-2018), Kronoberg (2006-2018), Norrbotten (2001-2018), Skåne (1989-2018), Stockholm (2003-2018), Södermanland (1992-2018), Uppsala (2005-2018), Västra Götaland (2000-2018), Värmland (2005-2018), Västerbotten (1991-2018), Västernorrland (2008-2018), Västmanland (2014-2018), Östergötland (1990-2018), and Örebro (2006-2018). The retrieval of data differs due to timing of digitalization of patient records. In 2018, 99% of the Swedish population lived in these 20 counties. For more information see *Sundquist, J., Ohlsson, H., Sundquist, K. et al. Common adult psychiatric disorders in Swedish primary care where most mental health patients are treated. BMC Psychiatry 17, 235 (2017).*

*The Population and Housing Censuses*

Every fifth year between 1960 and 1990 Sweden conducted censuses. These registers include among other things, the population's employment, the composition of households and housing. For more information, see *https://www.scb.se/hitta-statistik/statistik-efter-amne/befolkning/befolkningens-storlek-och-forandringar/registerbaserad-folk--och-bostadsrakning-census/*

*Prescribed Drug Register*

The Swedish Prescribed Drug Register started in July 2005 and includes all prescribed drugs being fetched at pharmacies, linked to personal numbers. For more information, see *https://www.socialstyrelsen.se/en/statistics-and-data/registers/national-prescribed-drug-register/*

*Cause of Death Register*

The Cause of Death Register includes all deaths occurring in Sweden from 1961 (including for Swedish citizens dying abroad) and is updated yearly. There is also a historical register between the years 1952 to 1960. For more information, see *https://www.socialstyrelsen.se/statistik-och-data/register/dodsorsaksregistret/*

*Criminal and Suspicion Register*

The Swedish Criminal Register and the Swedish Suspicion Register includes individual-level information on all committed crimes from 1973 and all suspicions of crimes related to an individual from 1998. For more information, see *https://polisen.se/lagar-och-regler/behandling-av-personuppgifter/polisens-register/*

Table 2 - Definition of disorders and variables

| Disorder/Variable | Registers Used | Definition |
| --- | --- | --- |
| Major Depression  (MD) | Hospital Discharge Register;  Outpatient Care Register;  Primary Care Data | Major Depression (MD) was identified in the Swedish medical registries by ICD codes: ICD8: 296.0, 296.2, 298.0, 300.4; ICD9: 296B, 298A, 300E; ICD10: F32, F33. |
| Mild/Moderate/Severe Major Depression | Hospital Discharge Register;  Outpatient Care Register;  Primary Care Data | Mild, Moderate and Severe Major Depression were identified in the Swedish medical registries by ICD10 codes: F32.0 and F33.0, F32.1 and F33.1, and F32.2 and F33.2, respectively. |
| Psychotic Major Depression | Hospital Discharge Register;  Outpatient Care Register;  Primary Care Data | Psychotic Major Depression was identified in the Swedish medical registries by ICD codes: ICD10: F32.3 and F33.3. |
| Prescribed antidepressants | Prescribed Drug Register | Prescribed antidepressants were identified in in the Prescribed Drug Register by all drugs in the Anatomical Therapeutic Chemical (ATC) Classification System group of N06A: Antidepressants. |
| Anxiety Disorder  (AD) | Hospital Discharge Register;  Outpatient Care Register;  Primary Care Data | Anxiety Disorder (AD) was identified in the Swedish medical registries by ICD codes: ICD-8: 300.0, 300.2; ICD-9: 300A, 300C; ICD-10: F40, F41 (except F41.2). |
| Generalized Anxiety Disorder  (GAD) | Hospital Discharge Register;  Outpatient Care Register;  Primary Care Data | Generalized Anxiety Disorder (GAD) was identified in the Swedish medical registries by ICD code: ICD10: F41.1. |
| Panic Disorder  (PD) | Hospital Discharge Register;  Outpatient Care Register;  Primary Care Data | Panic Disorder (PD) was identified in the Swedish medical registries by ICD code: ICD10: F41.0. |
| Phobic Anxiety Disorder  (Phobia) | Hospital Discharge Register;  Outpatient Care Register;  Primary Care Data | Phobic Anxiety Disorder (Phobia) was identified in the Swedish medical registries by ICD codes: ICD8: 300.2; ICD9: 300C; ICD10: F40. |
| Obsessive-Compulsive Disorder  (OCD) | Hospital Discharge Register;  Outpatient Care Register;  Primary Care Data | Obsessive-Compulsive Disorder (OCD) was identified in the Swedish medical registries by ICD codes: ICD8: 300.3; ICD9: 300D; ICD10: F42. |
| Bipolar Disorder  (BD) | Hospital Discharge Register;  Outpatient Care Register;  Primary Care Data | Bipolar Disorder (BD) was identified in the Swedish medical registries by ICD codes: ICD8: 296.1, 296.3, 296.8, 296.9, 298.1; ICD9: 296A, 296C, 296D, 296E, 296W, 298B; ICD10: F30, F31. |
| Attention Deficit Hyperactivity Disorder  (ADHD) | Hospital Discharge Register;  Outpatient Care Register;  Primary Care Data;  Prescribed Drug Register | Attention Deficit Hyperactivity Disorder (ADHD) was identified in the Swedish medical registries by ICD codes: ICD9: 314; ICD10: F90; in the Prescribed Drug Register by the drugs amfetamine (Anatomical Therapeutic Chemical (ATC) Classification System N06BA01), dexamfetamine (N06BA02), methylphenidate (N06BA04), and atomoxetine (N06BA09). |
| Post-Traumatic Stress Disorder  (PTSD) | Hospital Discharge Register;  Outpatient Care Register;  Primary Care Data | Post-Traumatic Stress Disorder (PTSD) was identified in the Swedish medical registries by ICD codes: ICD9: 308; ICD10: F43.0, F43.1, F62.0. |
| Alcohol Use Disorder  (AUD) | Hospital Discharge Register;  Outpatient Care Register;  Primary Care Data;  Prescribed Drug Register;  Cause of Death Register;  Criminal Register;  Suspicion Register | Alcohol Use Disorder (AUD) was identified in the Swedish medical and mortality registries by ICD codes: ICD8: 571.0, 291, 303; ICD9: V79B, 305A, 357F, 571A-D, 425F, 535D, 291, 303; ICD 10: E24.4, G31.2, G62.1, G72.1, I42.6, K29.2, K70, K85.2, K86.0, O35.4, F10.1-F10.9; in the Suspicion Register by codes 3005, 3201, which reflect crimes related to alcohol abuse; in the Criminal Register by references to laws covering crimes related to alcohol abuse (law 1951:649, paragraphs 4 and 4A and law 1994:1009, chapter 20, paragraphs 4 and 5) (only those individuals with at least two alcohol-related crimes or suspicion of crimes from both Crime Register and Suspicion Register were included); in the Prescribed Drug Register by the drugs disulfiram (Anatomical Therapeutic Chemical (ATC) Classification System N07BB01), acamprosate (N07BB03), and naltrexone (N07BB04). |
| Schizophrenia  (SZ) | Hospital Discharge Register;  Outpatient Care Register;  Primary Care Data | Schizophrenia (SZ) was identified in the Swedish medical registries by ICD codes: ICD8: 295.1, 295.2, 295.3, 295.6, 295.9; ICD9: 295B, 295C, 295D, 295G, 295X; ICD10: F20.0, F20.1, F20.2, F20.3, F20.5, F20.9. |

Table 3 – Trying out different sampling methodology of control probands

Different sampling methods were tried to determine its influence on HR of MD and tetrachoric correlations, and to confirm that individuals don’t get MD too early. The second line is the matching used in the present study. In the first line, we added proband birth year as a matching criteria, in the other four versions we went from incidence density sampling to cumulative density sampling, trying different cut-off values for lowest allowed age at onset of MD in control probands.

| Matching criteria | Controlling variables in Cox model | HR (95% CI) | Tetrachoric correlation: case/control status for proband vs sibling diagnosis | Tetrachoric correlation: proband diagnosis vs sibling diagnosis |
| --- | --- | --- | --- | --- |
| Sex  Birth year  Age at follow-up > age at MD for case  Age at MD > Age at MD for case | Sibling birth year  Sibling sex | 1.73  (1.71-1.75) | 0.202  (0.197-0.206) | 0.217  (0.213-0.222) |
| Sex  Age at follow-up > age at MD for case  Age at MD > Age at MD for case | Sibling birth year  Proband birth year  Sibling sex | 1.74  (1.72-1.76) | 0.201  (0.196-0.205) | 0.219  (0.214-0.223) |
| Sex  Age at follow-up > age at MD for case  Age at follow-up > 25  Age at MD > Age at MD for case  Age at MD > 25 | Sibling birth year  Proband birth year  Sibling sex | 1.75  (1.73-1.77) | 0.201  (0.196-0.205) | 0.218  (0.214-0.223) |
| Sex  Age at follow-up > age at MD for case  Age at follow-up > 30  Age at MD > Age at MD for case  Age at MD > 30 | Sibling birth year  Proband birth year  Sibling sex | 1.77  (1.75-1.80) | 0.209  (0.205-0.214) | 0.222  (0.217-0.226) |
| Sex  Age at follow-up > age at MD for case  Age at follow-up > 35  Age at MD > Age at MD for case  Age at MD > 35 | Sibling birth year  Proband birth year  Sibling sex | 1.78  (1.75-1.81) | 0.212  (0.207-0.216) | 0.222  (0.218-0.227) |
| Sex  Age at follow-up > age at MD for case  Age at follow-up > 40  Age at MD > Age at MD for case  Age at MD > 40 | Sibling birth year  Proband birth year  Sibling sex | 1.78  (1.74-1.81) | 0.219  (0.215-0.224) | 0.223  (0.219-0.228) |

Abbreviations: MD = major depression, HR = hazard ratio, CI = confidence interval

Table 4 – Details on R-packages used in statistical analyses

1. Therneau T. survival: A package for Survival Analysis in R. R package. 2022.

2. Wickham H. stringr: Simple, Consistent Wrappers for Common String Operations. R package. 2022.

3. Wickham H. ggplot2: Elegant Graphics for Data Analysis. New York, NY: Springer-Verlag; 2016.

4. Neuwirth E. RColorBrewer: ColorBrewer Palettes. R package. 2022.

5. Wickham H, Miller E, Smith D. haven: Import and Export 'SPSS', 'Stata' and 'SAS' Files. R package. 2022.

6. Wickham H, François R, Henry L, Müller K. dplyr: A Grammar of Data Manipulation. R package. 2023.

7. Dowle M, Srinivasan A. data.table: Extension of ‘data.frame’. R package. 2022.

8. Fox J. polycor: Polychoric and Polyserial Correlations. R package. 2022.

Table 5 – The impact of major depression (MD) proband status on the hazard ratio in siblings for MD, anxiety disorders, obsessive-compulsive disorder, bipolar disorder, attention deficit hyperactivity disorder, post-traumatic stress disorder, alcohol use disorder, and schizophrenia.

| Diagnosis | HR | 95% CI^a^ |
| --- | --- | --- |
| MD | 1.74 | (1.72-1.76)^****^ |
| GAD | 1.79 | (1.74-1.85)^****^ |
| PD | 1.68 | (1.64-1.72)^****^ |
| Phobia | 1.71 | (1.65-1.76)^****^ |
| OCD | 1.72 | (1.65-1.80) ^****^ |
| BD | 1.78 | (1.70-1.85)^****^ |
| ADHD | 1.82 | (1.76-1.88)^****^ |
| PTSD | 1.62 | (1.59-1.66)^****^ |
| AUD | 1.64 | (1.60-1.68)^****^ |
| SZ | 1.42 | (1.30-1.54)^****^ |
| ^a^ Significance levels for p-values: *<.05, **<.01, ***<.001, ****<.0001 | | |

Abbreviations: HR = hazard ratio, CI = confidence interval, MD = major depression, GAD = generalized anxiety disorder, PD = panic disorder, Phobia = phobic anxiety disorder, OCD = obsessive- compulsive disorder, BD = bipolar disorder, ADHD = attention deficit hyperactivity disorder, PTSD = post-traumatic stress disorder, AUD = alcohol use disorder, SZ = schizophrenia

Analyses controlled for sex and birth year of the siblings. P-values based on the interaction between clinical features and psychiatric diagnoses in sibling. All measured from age 10 and onwards for reasons of stability.

Table 6 - Linear effects of younger age at onset and number of episodes of MD probands on the hazard ratio in siblings for MD, anxiety disorders, obsessive-compulsive disorder, bipolar disorder, attention deficit hyperactivity disorder, post-traumatic stress disorder, alcohol use disorder, and schizophrenia.

|  | Younger age at onset | | No. of episodes | |
| --- | --- | --- | --- | --- |
| Diagnosis | HR | 95% CI^a^ | HR | 95% CI^a^ |
| MD | 1.07 | (1.06-1.08)^****^ | 1.05 | (1.05-1.06)^****^ |
| GAD | 1.06 | (1.04-1.08)^****^ | 1.04 | (1.03-1.05)^****^ |
| PD | 1.05 | (1.04-1.07)^****^ | 1.04 | (1.03-1.05)^****^ |
| Phobia | 1.07 | (1.05-1.10)^****^ | 1.05 | (1.03-1.06)^****^ |
| OCD | 1.09 | (1.06-1.12)^****^ | 1.05 | (1.03-1.07)^****^ |
| BD | 1.08 | (1.05-1.12)^****^ | 1.04 | (1.02-1.05)^****^ |
| ADHD | 1.04 | (1.02-1.07)^***^ | 1.04 | (1.02-1.05)^****^ |
| PTSD | 1.07 | (1.06-1.09)^****^ | 1.03 | (1.02-1.04)^****^ |
| AUD | 1.07 | (1.05-1.08)^****^ | 1.03 | (1.02-1.04)^****^ |
| SZ | 1.05 | (0.99-1.12) | 1.06 | (1.02-1.09)^**^ |
| ^a^ Significance levels for p-values: *<.05, **<.01, ***<.001, ****<.0001 | | | | |

Abbreviations: HR = hazard ratio, CI = confidence interval, MD = major depression, GAD = generalized anxiety disorder, PD = panic disorder, Phobia = phobic anxiety disorder, OCD = obsessive- compulsive disorder, BD = bipolar disorder, ADHD = attention deficit hyperactivity disorder, PTSD = post-traumatic stress disorder, AUD = alcohol use disorder, SZ = schizophrenia

Analyses controlled for sex and birth year of the siblings. P-values based on the interaction between clinical features and psychiatric diagnoses in sibling. Only linear effect was analyzed to enable comparison between psychiatric disorders. Younger age at onset is measured as a continuous variable in five-year units.

Table 7 - Influence of probands comorbidities on siblings’ psychiatric disorders

|  | Proband MD without proband screening | | Proband MD with proband screening on having disorder and MD | | |
| --- | --- | --- | --- | --- | --- |
| Offspring diagnosis | HR | 95% CI^a^ | Number of probands diagnosed with disorder and MD | HR | 95% CI^a^ |
| GAD | 1.79 | (1.74-1.85)^****^ | 31,168 | 1.69 | (1.63-1.74)^****^ |
| PD | 1.68 | (1.64-1.72)^****^ | 42,192 | 1.50 | (1.47-1.54)^****^ |
| Phobia | 1.71 | (1.65-1.76)^****^ | 23,405 | 1.59 | (1.54-1.65)^****^ |
| OCD | 1.72 | (1.65-1.80)^****^ | 12,863 | 1.62 | (1.55-1.69)^****^ |
| ADHD | 1.82 | (1.76-1.88)^****^ | 25,596 | 1.53 | (1.48-1.58)^****^ |
| PTSD | 1.62 | (1.59-1.66)^****^ | 52,823 | 1.49 | (1.45-1.53)^****^ |
| AUD | 1.64 | (1.60-1.68)^****^ | 25,661 | 1.43 | (1.39-1.46)^****^ |
| ^a^ Significance levels for p-values: *<.05, **<.01, ***<.001, ****<.0001 | | | | | |

Correction type 1: Exclusion of the case probands (along with their matched control proband) having MD and the disorder of interest.

Correction type 2: Controlling for the comorbid disorder in the Cox regression. A dummy variable included which is the same for both case and control probands.

|  | Proband MD without proband screening | | Proband MD with proband screening on having disorder and MD | | |
| --- | --- | --- | --- | --- | --- |
| Offspring diagnosis | HR | 95% CI^a^ | Number of probands diagnosed with disorder and MD | HR | 95% CI^a^ |
| GAD | 1.79 | (1.74-1.85)^****^ | 31,168 | 1.69 | (1.64-1.75)^****^ |
| PD | 1.68 | (1.64-1.72)^****^ | 42,192 | 1.55 | (1.51-1.58)^****^ |
| Phobia | 1.71 | (1.65-1.76)^****^ | 23,405 | 1.60 | (1.55-1.65)^****^ |
| OCD | 1.72 | (1.65-1.80)^****^ | 12,863 | 1.64 | (1.57-1.71)^****^ |
| ADHD | 1.82 | (1.76-1.88)^****^ | 25,596 | 1.58 | (1.53-1.64)^****^ |
| PTSD | 1.62 | (1.59-1.66)^****^ | 52,823 | 1.52 | (1.48-1.55)^****^ |
| AUD | 1.64 | (1.60-1.68)^****^ | 25,661 | 1.48 | (1.44-1.52)^****^ |
| ^a^ Significance levels for p-values: *<.05, **<.01, ***<.001, ****<.0001 | | | | | |

Abbreviations: HR = hazard ratio, CI = confidence interval, MD = major depression, GAD = generalized anxiety disorder, PD = panic disorder, Phobia = phobic anxiety disorder, OCD = obsessive- compulsive disorder, ADHD = attention deficit hyperactivity disorder, PTSD = post-traumatic stress disorder, AUD = alcohol use disorder
